# Photodynamic Therapy Can Induce a Protective Innate Immune Response against Murine Bacterial Arthritis *via* Neutrophil Accumulation

**DOI:** 10.1371/journal.pone.0039823

**Published:** 2012-06-26

**Authors:** Masamitsu Tanaka, Pawel Mroz, Tianhong Dai, Liyi Huang, Yuji Morimoto, Manabu Kinoshita, Yasuo Yoshihara, Koichi Nemoto, Nariyoshi Shinomiya, Suhji Seki, Michael R. Hamblin

**Affiliations:** 1 Wellman Center for Photomedicine, Massachusetts General Hospital, Boston, Massachusetts, United States of America; 2 Department of Dermatology, Harvard Medical School, Boston, Massachusetts, United States of America; 3 Department of Orthopedic Surgery, National Defense Medical College, Tokorozawa, Saitama, Japan; 4 Department of Integrated Physiology and Bio-Nano Medicine, National Defense Medical College, Tokorozawa, Saitama, Japan; 5 Department of Immunology and Microbiology, National Defense Medical College, Tokorozawa, Saitama, Japan; 6 Harvard-MIT Division of Health Sciences and Technology, Cambridge, Massachusetts, United States of America; Wayne State University, United States of America

## Abstract

**Background:**

Local microbial infections induced by multiple-drug-resistant bacteria in the orthopedic field can be intractable, therefore development of new therapeutic modalities is needed. Photodynamic therapy (PDT) is a promising alternative modality to antibiotics for intractable microbial infections, and we recently reported that PDT has the potential to accumulate neutrophils into the infected site which leads to resolution of the infection. PDT for cancer has long been known to be able to stimulate the innate and adaptive arms of the immune system.

**Methodology/Principal Findings:**

In the present study, a murine methicillin-resistant *Staphylococcus aureus* (MRSA) arthritis model using bioluminescent MRSA and polystyrene microparticles was established, and both the therapeutic (Th-PDT) and preventive (Pre-PDT) effects of PDT using methylene blue as photosensitizer were examined. Although Th-PDT could not demonstrate direct bacterial killing, neutrophils were accumulated into the infectious joint space after PDT and MRSA arthritis was reduced. With the preconditioning Pre-PDT regimen, neutrophils were quickly accumulated into the joint immediately after bacterial inoculation and bacterial growth was suppressed and the establishment of infection was inhibited.

**Conclusions/Significance:**

This is the first demonstration of a protective innate immune response against a bacterial pathogen produced by PDT.

## Introduction

Antibiotic therapy is still a mainstay of a treatment for microbial infections in orthopedic fields. However, treatments for orthopedic infectious disease, e.g. arthritis or osteomyelitis can be problematic [1], [2] due to various reasons: bone, cartilage and joint are naturally aseptic: the blood supply to the tissues is lower: the frequent use of artificial biomaterial implants made from metal or resin easily facilitates the formation of a biofilm and reduces the response to antibiotics. The occurrence of post-operative surgical-site infections (SSI) in bone and joint surgery has gradually decreased due to the widespread use of sterile operation procedures and adequate use of antibiotic therapy and recent data shows that the occurrence of SSI after total knee arthroplasty (TKA) is less than 1% [1], [2], for example. However, once SSI does occur after orthopedic surgery, adhesive biofilms are easily formed on the surface of metal or resin biomaterials and the infection often becomes resistant to conventional antibiotic therapy.

Furthermore, SSI caused by multidrug-resistant bacteria such as methicillin-resistant *Staphylococcus aureus* (MRSA) continues to be a serious problem. Bone or joint infections caused by multidrug-resistant bacteria are extremely intractable; therefore patients who suffer from these infections have to undergo invasive treatments such as surgical excision and curettage or continuous irrigation in addition to long-term antibiotic administration, resulting in prolonged hospitalization and diminution in the quality of life [3], [4]. Excessive use of antibiotics encourages the spread of multidrug-resistant bacteria, therefore new therapeutic modalities as an alternative to antibiotics are needed. Although antibiotic treatment has also been used for a prevention of SSI, the preventive effect depends on the sensitivity of bacteria to antibiotics and therefore SSI with multidrug-resistant bacteria such as MRSA could not be eliminated.

In recent years, photodynamic therapy (PDT) has been examined as an alternative approach to treat local microbial infections. PDT originated from the development of photodynamic agents (photosensitizers, PS) for clinical use in 1960s, and has been clinically applied to cancer treatment e.g. for early stage lung cancer [5]. PDT is considered as a non-invasive therapeutic modality for malignant tumors. PS accumulated into the tumor cells is activated by visible light, and the activated PS induces the generation of reactive oxygen species, which damage the unwanted tissues or cells [6]–[10]. PDT has been clinically applied to the treatment for early stage pulmonary, gastric and esophageal carcinoma, and has been examined for an application to other diseases such as retinal diseases [11], [12] or cardiovascular disorders [13], [14]. Since the 1990s, PDT has attracted considerable attention as a possible alternative approach for local microbial infection, and there have been an increasing number of reports of applications of PDT for infections with multidrug-resistant bacteria [15]–[20]. There have been no reports that described PDT-resistance of bacteria, thus the application PDT for localized microbial infections could be a new promising modality for bone and joint infections regardless of the antibiotic sensitivity or multi-drug resistance, therefore diminution of the patient’s quality of life could be avoided. However, although favorable results of *in vitro* PDT for cultured bacteria have been described in many reports, good results *in vivo* in animal models of localized infections have only been described in a few reports especially in the area of bone and joint infectionss [19], [20].

PDT is known to stimulate both the innate and adaptive arms of the immune system and this aspect has been intensively investigated as part of the anti-cancer effect of PDT [21]. The acute inflammatory effects of PDT produce cytokines and chemokines that can attract and activate neutrophils [22], macrophages [23] and dendritic cells [24]. Furthermore damage-associated molecular patterns (DAMPS) can be produced by PDT-mediated tissue damage that can produce further immune activation [25]. In some circumstances this activation of innate immune cells leads to production of tumor specific T-cells and B-cells and an antigen-specific immune response develops [26]. Under favorable conditions (e.g. the tumor expresses a tumor-rejection antigen) this immune response can lead to rejection of tumor rechallenge [27], cure of metastatic tumors [28] and even regression of distant established tumors [26]. However to our our knowledge there have been no reports of activation of the immune system towards a microbial pathogen after PDT.

Bacterial phagocytosis by innate immune cells such as neutrophils plays a crucial role in the elimination of invading bacteria, especially *Staphylococcus aureus* [29]–[31]. Malfunction of the phagocytic immune system therefore renders the host susceptible to bacterial infections [32]. This fact is crucial when considering the clinical situation of antimicrobial treatment. If a treatment impairs the function of phagocytes in combating microbial infection, the efficacy of the antimicrobial treatment might be reduced, resulting in deterioration and prolongation of the infection. Therefore, the undesirable influences of PDT, possible cytocidal effects on phagocytes in particular, should be minimized when considering the clinical application of PDT against local microbial infection.

We have investigated the therapeutic effect of PDT for a murine MRSA arthritis model focusing on the role of local host defense mechanisms in the PDT response [33]–[35]. We recently demonstrated that PDT using Photofrin could damage neutrophils as well as bacteria *in vitro* [33], and *in vivo* PDT using Photofrin for murine MRSA-induced arthritis showed a pronounced biphasic light dose response [34]. Light doses that were too low and also light doses that were too high were ineffective and there was an optimum fluence to give the best antibacterial effect. PDT at a fluence that could accumulate neutrophils into the infectious site led to a favorable resolution of infection. Additionally, we demonstrated that the PDT damage to neutrophils was minimized when methylene blue or toluidine blue-O was used as a PS [35]. Methylene blue, in particular, had the best selectivity for bacteria while preserving neutrophils, and additionally has a long wavelength absorbance peak (664 nm), which could help to treat for deep regional infections [36].

Based on these results, in this study, we selected methylene blue as the optimal PS for antimicrobial PDT and examined the *in vivo* therapeutic effect of PDT in a murine MRSA arthritis model in the knee joint, focusing on the effects on host defense mechanisms such as neutrophils (therapeutic PDT: Th-PDT). Furthermore, to examine whether PDT could prevent microbial infection by making use of its ability to stimulate host defense mechanisms, we performed a preconditioning regimen of PDT before infection (preventive PDT: Pre-PDT).

Prior to those examinations, we tried to resolve several problems encountered in the previous study [33], [34]. The problems were as follows: firstly, the number of remaining viable MRSA in the joint was decreased once the synovial fluid was collected, therefore the time course of the severity of the infection could not be precisely evaluated with the method of direct counting of colony-forming units (CFU) of the MRSA in the synovial fluid collected from the knee joint; secondly, even the control infections tended to resolve, therefore the PDT effect could not be clearly demonstrated. To resolve the first problem, we used luciferase-expressing bioluminescent MRSA [18], [37] and *in vivo* bioluminescence imaging and measured the intensity of bioluminescence to demonstrate longitudinal progress of infection in the same mouse. To resolve the second problem MRSA was inoculated with artificial resin microparticles into the mouse knee joint to enable to establish biofilm formation that leads to severe and intractable arthritis.

## Results

### Time Courses of a Mouse Model Using Resin Microparticle (MP+ Group) and a Model without Resin Microparticle (MP- Group)

**Figure 1:**
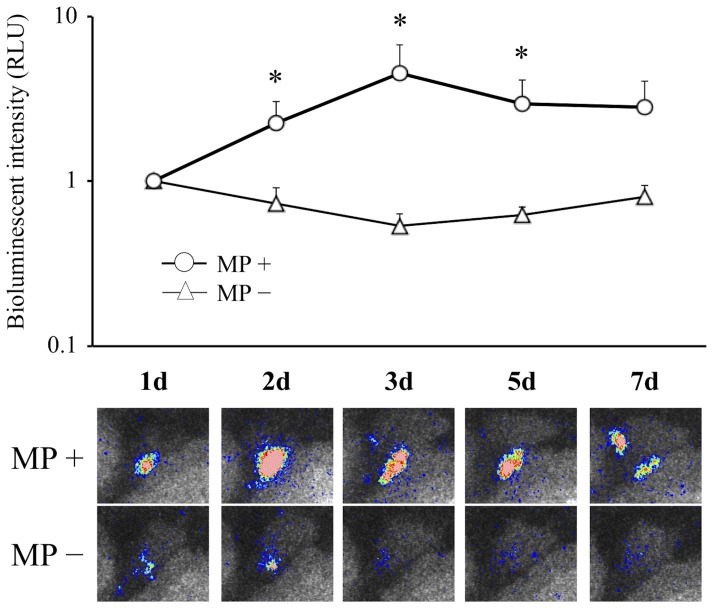
MRSA arthritis model followed by bioluminescence imaging. (a) Time course of the bioluminescent signal of MRSA arthritis models. (b) Images of bioluminescence in the model using microparticle (MP+ group) and in the model without microparticle (MP- group). *n* = 5 each. *P<0.05.

**Figure 2:**
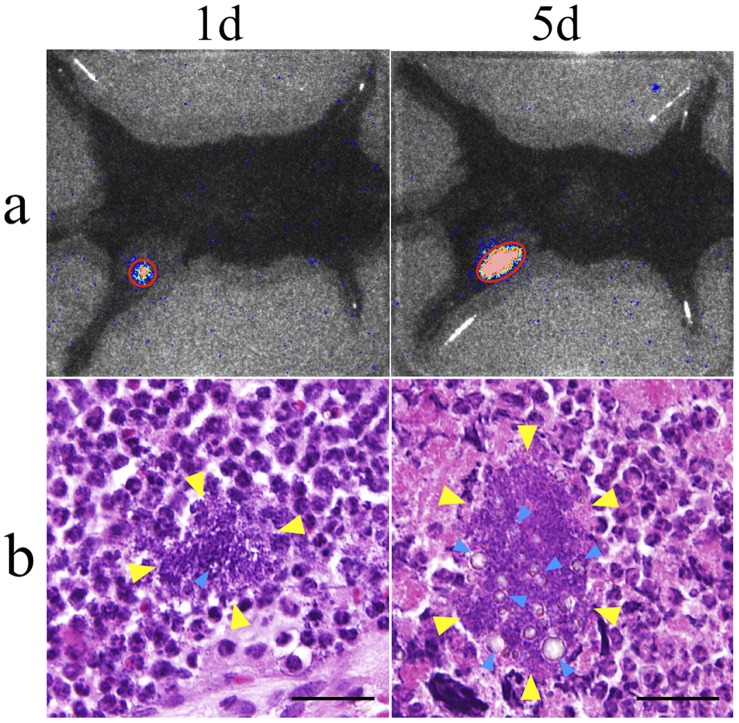
MRSA arthritis model at days 1 and 5. a : Images of the bioluminescent MRSA-infected left knee joint of the MRSA arthritis model (PS-IR- group). **b**: Histopathological images of MRSA colony with biofilm formation around the polystyrene particles. Blue arrows: resin microparticles, yellow arrows: boundary of the MRSA colony. Bar = 30 µm.

Bioluminescence from the mouse knee joint was observed in each MP+ and MP- group. The bioluminescent intensity in the MP+ group was significantly higher than that in the MP- group until 7 days after MRSA inoculation (Fig. 1). Histopathologically, a bacterial colony was observed to be formed around the resin microparticle, and leukocytes were outside of the boundary of the infection and did not permeate into the border, indicating localized bacterial growth with biofilm formation (Fig. 2). According to these results, we used the MP+ group model as a chronic bacterial arthritis model in all the following experiments.

### The Effect of Therapeutic PDT (Th-PDT)

**Figure 3:**
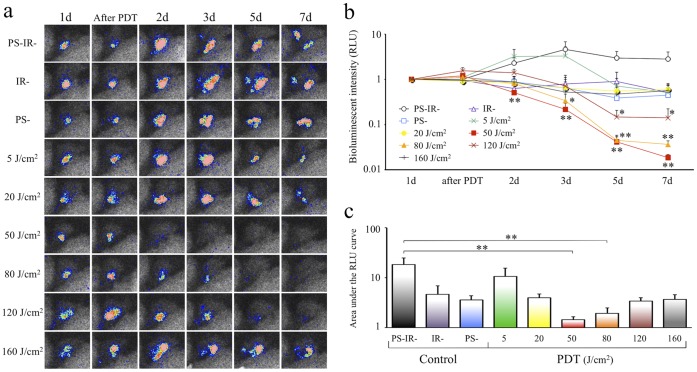
Effect of PDT doses on MRSA arthritis. a : Time course series of bioluminescent images after therapeutic PDT (Th-PDT) in each irradiation energy group. **b**: A line graph of time courses of bioluminescent intensity after Th-PDT in each irradiation energy group. **c**: Comparison of the area under the RLU curve (AUC) indicated in b. *n* = 5 each. *P<0.05, **P<0.01.

The time course series of bioluminescent images of the intra-articular *lux*-MRSA in each group was used for the evaluation of the therapeutic effect of PDT. Bioluminescent intensity was not significantly decreased in any of the control groups. On the other hand, the bioluminescent intensity was significantly decreased in the 50, 80 and 120 J/cm^2^ group (Fig. 3a). Especially in the 50 and 80 J/cm^2^ groups, the signal intensity was decreased at time points up to 7 days after infection, and the 50 J/cm^2^ group showed the lowest value throughout the period (Fig. 3a and 3b). The bioluminescent intensity was not decreased in the other Th-PDT groups. However, contrarily to our expectation, the bioluminescent intensity was not decreased immediately after PDT compared to that before PDT in each group (Fig. 3a and 3b).

In the comparison of the mean area under the curve (AUC), the value of the 50 J/cm^2^ group was the lowest and the antimicrobial effect was lessened when the light dose was either lower or higher than 50 J/cm^2^, which showed a biphasic light dose response curve.

The mean values of the AUC of the leg function score were higher in the PDT groups from 20 to 120 J/cm^2^ than that in the PS-IR- group (Suppl. data Figs S1 a and S1 b). The AUC of the erosion score were also higher in the PDT groups from 20 to 120 J/cm^2^ than that in the PS-IR- group (Suppl. data Figs S1c and S1d). Both the leg function score and the erosion score showed a biphasic light dose response curve, indicating that Th-PDT in at the appropriate dose could prevent function loss of the infected knee joint.

### Histopathological Changes in the Infected Knee Joints Before and After Therapeutic PDT (Th-PDT)

**Figure 4:**
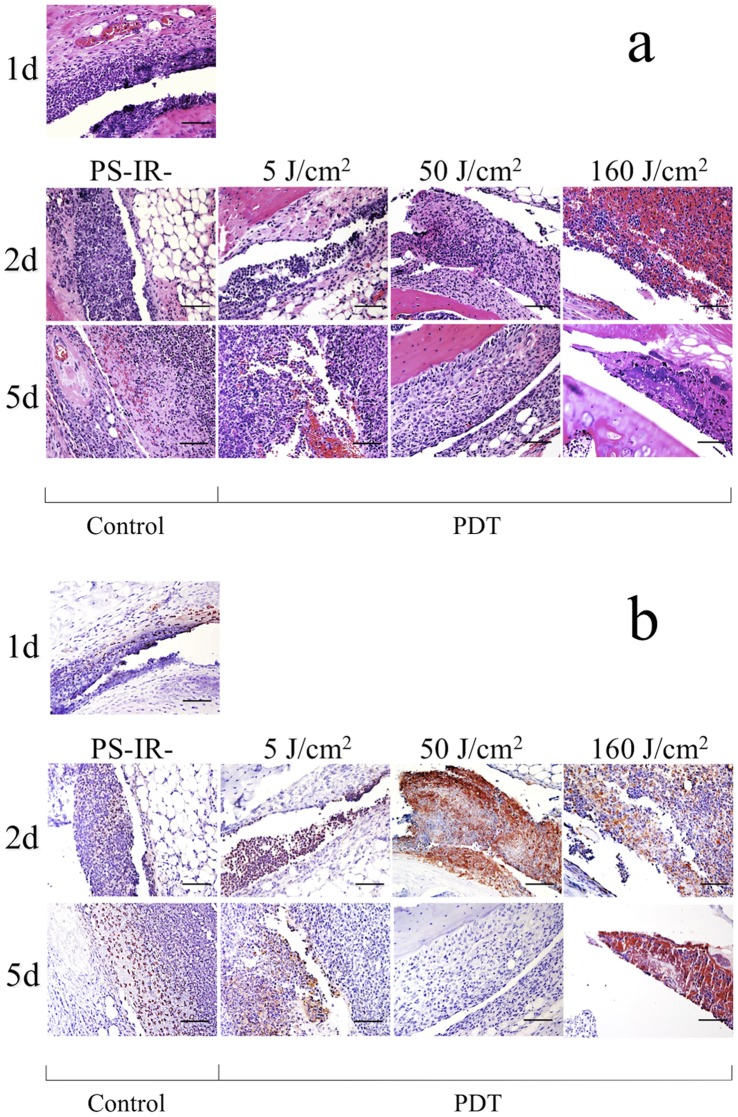
Histology of knees from mice with MRSA arthritis. Histopathological images of the intraarticular tissues of the knee joint in each Th-PDT group (PS-IR-, 5, 50 and 160 J/cm^2^). **a**: Hematoxilin-eosin staining, **b**: immunostaining using an anti-GR-1 antibody. Bar = 50 µm.

Histopathological examination was performed for the evaluation of neutrophil accumulation into the infected site after PDT. One day after *lux*-MRSA inoculation, although nucleated cells were accumulated into the joint space and synovium (Fig. 4a), only a few GR-1-positive cells (neutrophils) were seen (Fig. 4b). In the PS-IR- group, no remarkable change in the number of GR-1-positive cells was seen at 2d or 5d, and the cells still remained in the synovium at 5d (Fig. 4a and 4b). In the 5 J/cm^2^ group, more GR-1-positive cells were seen at 2d, and the cells still remained in the hyperplastic synovium at 5d (Fig. 4a and 4b). In the 50 J/cm^2^ group, a lot of GR-1-positive cells were seen in the joint space and synovium at 2d. The synovium was hyperplastic at 5d; however, few GR-1-positive cells were seen in the synovium at 5d (Fig. 4a and 4b). In the 160 J/cm^2^ group, synovial microvessels were damaged and a large number of red blood cells were seen in the joint space at 2d, indicating tissue damage by PDT (Fig. 4a). At 5d, a GR-1 positive region was observed; however, it was a cell accumulation surrounding a bacterial colony and a resin microparticle (Fig. 4b). These data showed that PDT at the correct dose could accumulate neutrophils into the infected site, and correlated with the resolution of the infection.

### The Effect of Therapeutic PDT (Th-PDT) in the Neutrophil-depletion Model

**Figure 5:**
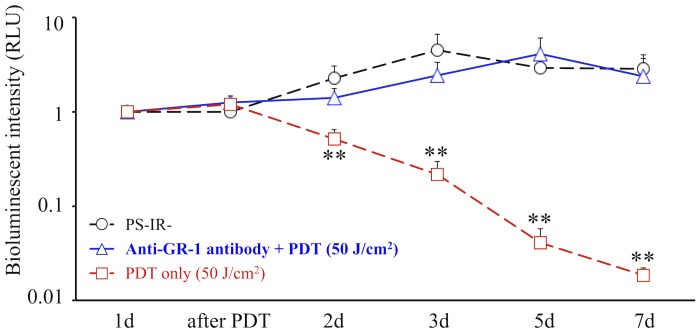
Effect of anti-GR-1 antibody. Comparison of time courses of the bioluminescent intensity in the PS-IR- group, the anti-GR-1 antibody + Th-PDT (50 J/cm^2^) group and the Th-PDT (50 J/cm^2^) group. *n* = 5 each. **P<0.01.

In the 50 J/cm^2^ group with neutrophil-depletion using intravenous anti-GR-1 antibody, the consequent bioluminescent intensity was maintained at high levels until 7d, which was similar to that in the PS-IR- group (Fig. 5), indicating that therapeutic effect of PDT was lost when neutrophils were depleted using intravenous anti-GR-1 antibody.

### The Effect of Preventive PDT (Pre-PDT) on Suppression of the Infection

**Figure 6:**
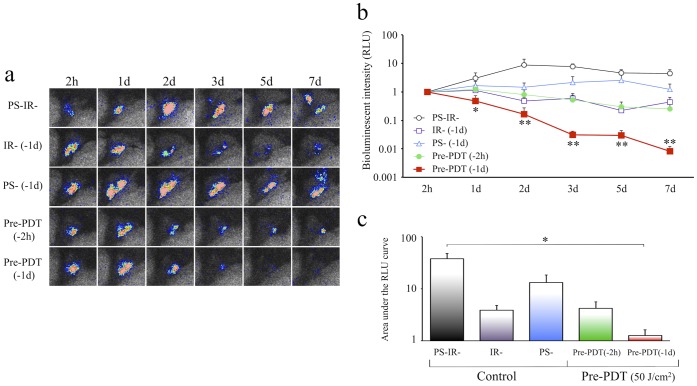
Effect of Pre-PDT on MRSA arthritis. a : Time course series of bioluminescent images after preventive PDT (Pre-PDT) in each group. **b**: A line graph of time courses of bioluminescent intensity after Pre-PDT in each group. **c**: Comparison of the area under the RLU curve (AUC) of the data indicated in b. *n* = 5 each. *P<0.05, **P<0.01.

A suppressive effect on *lux*-MRSA arthritis was seen neither in the control groups nor in the short interval Pre-PDT (−2 h) group. On the other hand, the bioluminescent intensity was significantly decreased in the long interval Pre-PDT (−1d) group, indicating a suppression of bacterial growth and an inhibition of infection when PDT was carried out one day before infection (Fig. 6a–c). The AUC of both the leg function score and the erosion score in the Pre-PDT (−1d) group were significantly higher than that in the control groups (Suppl. data Figs S2 a-d), indicating that the long interval Pre-PDT could prevent function loss of the infected knee joint.

### Histopathological Changes in Infected Knee Joints Before and After Preventive PDT (Pre-PDT)

**Figure 7:**
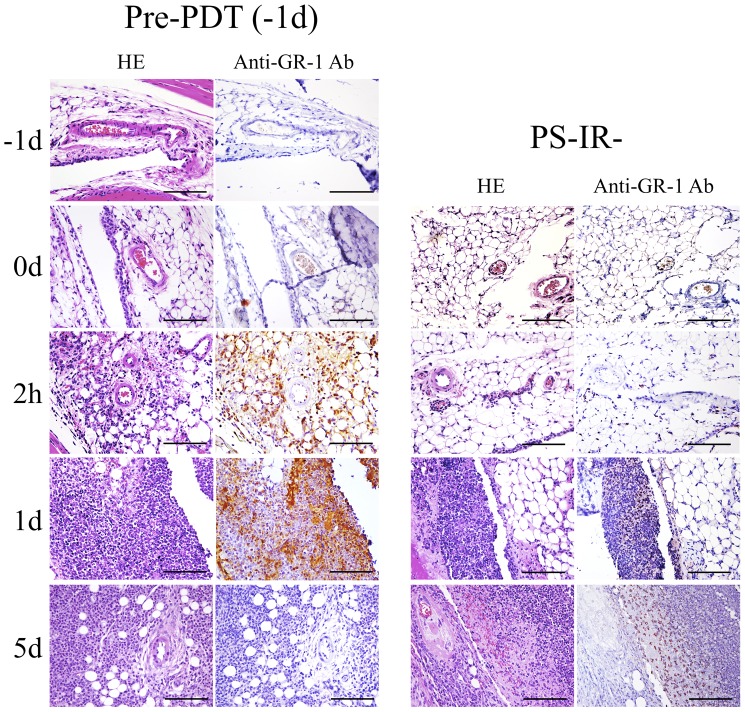
Histology of knees from Pre-PDT treated mice. Histopathological images of the intraarticular tissues of the knee joint in the preventive PDT (Pre-PDT) group and the PS-IR- group (hematoxylin-eosin staining and immunostaining using an anti-GR-1 antibody). Bar = 50 µm.

Histopathological examination was performed to evaluate when neutrophils were accumulated after Pre-PDT. In the Pre-PDT (−1d) group, the synovium and microvessels remained intact and no accumulation of GR-1-positive cells (neutrophils) was seen in the knee joint immediately after Pre-PDT and the next day (just before *lux*-MRSA inoculation). However, many GR-1-positive cells were accumulated and migrated into the tissues around the microvessels only 2 hours after *lux*-MRSA inoculation, and further large numbers of GR-1 positive cells were accumulated in the joint space 1 day after the MRSA inoculation (at 1d). Five days after inoculation, only a few GR-1 positive cells were seen in the hyperplastic synovium or joint space, indicating the suppression of infection (Fig. 7). On the other hand, in the PS-IR- group, few GR-1-positive cells had migrated into the tissue around microvessels at 2 h. Although many GR-1-positive cells were seen in the knee joint at 1d, their number was still less than that in the Pre-PDT (−1d) group. The synovium was hyperplastic and many GR-1-positive cells were seen even 5 d after the MRSA inoculation, indicating the protraction of the course of arthritis (Fig. 7).

### The Effect of Preventive PDT (Pre-PDT) in the Neutrophil-depletion Model

**Figure 8:**
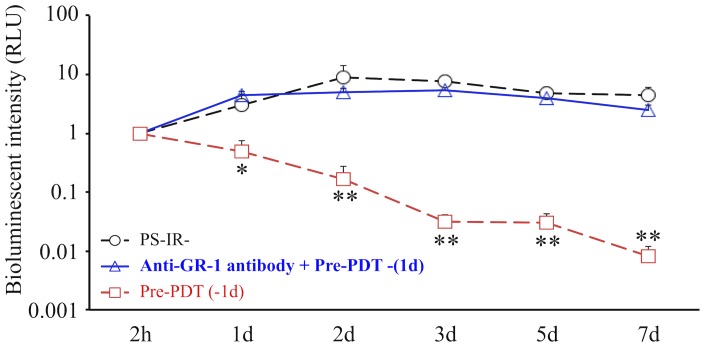
Effect of anti-GR-1 antibody on Pre-PDT response. Comparison of time courses of bioluminescent intensity in the PS-IR- group, the anti-GR-1 antibody + Pre-PDT (−1d) group and the Pre-PDT (−1d) group. *n* = 5 each. *P<0.05, **P<0.01.

In the Pre-PDT (−1d) group with neutrophil-depletion using intravenous anti-GR-1 antibody, the bioluminescent intensity was maintained at high levels until 7d, which was similar to that in the PS-IR- group (Fig. 8), indicating that the suppressive effect of Pre-PDT on bacterial growth was lost when neutrophils were decreased using neutralizing anti-GR-1 antibody.

### Time Courses of Intra-articular Leukocyte Counts After Preventive PDT (Pre-PDT)

**Figure 9:**
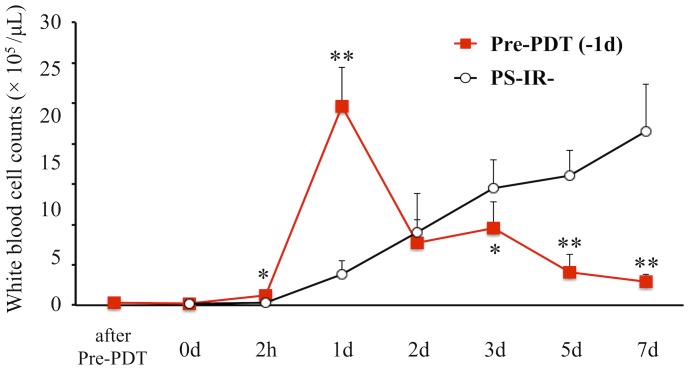
Time course of intrarticular leukocytes. Comparison of time courses of intraarticular leukocyte counts in the Pre-PDT (−1d) group and the PS-IR- group. *n* = 5 each. *P<0.05, **P<0.01.

In the Pre-PDT (−1d) group, intra-articular leukocyte counts were not increased immediately after Pre-PDT (at −1d) and 1 day after Pre-PDT (at 0d). However, counts were gradually increased from 2 hours after *lux*-MRSA inoculation and reached an extremely high level 1 day after the MRSA inoculation, then gradually decreased (Fig. 9), correlating with the results shown in Fig. 8. On the other hand, in the PS-IR- group, although intra-articular leukocytes were not increased 2 hours after MRSA inoculation (at 2h), then gradually increased from 1 day after the inoculation for the whole period of study until the last observation, indicating the protraction of the course of arthritis (Fig. 9).

### Influences of Chemotactic Factors on the Effect of Preventive PDT (Pre-PDT)

**Figure 10:**
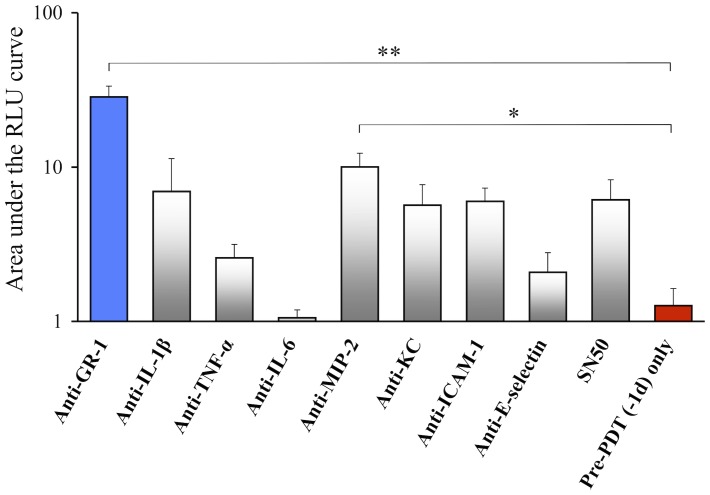
Effect of blocking antibodies on Pre-PDT response. Comparison of the area under the RLU curve (AUC) in the Pre-PDT (−1d) + each antibody groups and the Pre-PDT (−1d) group. These values were determined from the time courses shown in Figure S3. *n* = 5 each. *P<0.05, **P<0.01.

The suppressive effect of Pre-PDT for the development of infection was abrogated by the administration of neutralizing antibodies for chemotactic factors that are associated with the neutrophil accumulation into the infectious site, except for an anti-interleukin-6 (IL-6) antibody (Supp. data 3). In the comparison of AUC, anti-macrophage-inflammatory-protein 2 (anti-MIP-2) antibody significantly reduced the Pre-PDT effect (Fig. 10). The other antibodies showed a tendency to reduce the Pre-PDT effect; however, their AUC values were not significantly different from that in the Pre-PDT (−1d) group (Fig. 10). SN50 [38], which is an inhibitor of nuclear factor-kappa B (NF-κB, a transcription factor which is closely related to the initial inflammatory reaction), also showed a tendency to reduce the Pre-PDT effect; however, the AUC value was not significantly different from that in the Pre-PDT (−1d) group (Fig. 10).

## Discussion

We established a murine chronic MRSA arthritis model using a combination of bioluminescent MRSA and resin microparticles, which allowed sequential non-invasive optical evaluation of the course of the infection in an individual mouse and enabled us to carry out a detailed examination of the PDT effects in an efficient manner.

Using appropriate light doses, therapeutic PDT (Th-PDT) successively reduced the bioluminescent intensity of *lux*-MRSA in the knee joint from day of PDT for the succeeding 7 days, and gave a therapeutic effect with 2-log10 reduction of bacterial bioluminescence signal. The effective light dose ranged from 50 to 80 J/cm^2^, indicating that the therapeutic window of PDT using methylene blue was broader than that of PDT using Photofrin [34]. The administration of anti-GR-1 (anti-neutrophil) antibody eliminated the therapeutic effect of PDT, indicating that the therapeutic PDT using methylene blue exerted a therapeutic effect for bacterial infection *via* the attraction and accumulation of neutrophils into the infected region.

The bioluminescent intensity was not decreased immediately after Th-PDT using any of the light doses, indicating that Th-PDT did not exert a direct bacterial killing effect as expected. This finding might be due to biofilm formation on the surface of the resin microparticles that inhibited the binding of methylene blue molecules to *lux*-MRSA cells and therefore led to the failure to exert a direct bacterial killing. However, it has been reported that PDT using methylene blue can disrupt biofilms [39]–[41] and this might enable accumulated neutrophils to penetrate into the biofilm and to kill the bacteria [42]. The therapeutic effect on MRSA arthritis we hypothesized to be due to phagocytosis of the bacteria by the accumulated and activated neutrophils stimulated by PDT rather than *via* a direct bactericidal PDT killing.

These findings indicated that PDT using methylene blue could strongly activate innate host defense mechanisms against a microbial infection. Therefore, we hypothesized that PDT might suppress bacterial growth and inhibit the establishment of infection when performed as a protective pre-conditioning regimen. PDT was conducted on a murine normal knee joint (preventive PDT: Pre-PDT), and we examined the protective effect of Pre-PDT on the *in vivo* development of infection. Pre-PDT performed on normal tissue 1 day before the bacterial inoculation strongly inhibited the subsequent bacterial growth and suppressed the development of the infection, and moreover the effect was eliminated by administration of an intravenous anti-neutrophil antibody, indicating that neutrophil activity played an important role in the protection against infection by Pre-PDT as well as the resolution of infection by the Th-PDT regimen.

The histopathological examination and the time course of intra-articular leukocyte counts showed that although neither neutrophil accumulation nor any other noticeable changes were seen in the knee joint at 1 day after Pre-PDT, before bacteria were inoculated. However neutrophils immediately migrated into the tissue around microvessels and the number of leukocytes, mainly neutrophils, in the knee joint began to increase within 2 hours after bacterial inoculation. Generally, neutrophil accumulation into the regions of bacterial infection needs at least 6 hours from bacterial invasion because time is needed for the expression of chemotactic factors on vascular endothelium and surrounding tissues [43], [44]. Since Pre-PDT carried out 24 hours previously (but not 2 hours previously) enabled neutrophils to accumulate and migrate into the infectious site within 2 hours after bacterial inoculation it is suggested that a certain preparatory state of neutrophil priming was achieved. Further investigation of the mechanisms of this neutrophil priming should be performed.

We performed Pre-PDT with a set of neutralizing antibodies for chemotactic factors, which are responsible for neutrophil activation and accumulation. All antibodies, except for the anti-IL-6 antibody, abrogated the protective effect of Pre-PDT on bacterial growth; however, the extent of abrogation was limited compared to that found in the group using anti-GR-1 and MIP-2 antibodies. These results suggested that all the tested chemotactic factors (except for IL-6) were involved in the effect of Pre-PDT to stimulate innate immunity. In other words, Pre-PDT might activate the whole sequential cascade for the accumulation, migration and activation of neutrophils into the local infectious site. MIP-2 in particular, is expressed by macrophages and fibroblasts [45], therefore the initial mediators of the neutrophil-mediated protective action of Pre-PDT might be macrophages or synovial fibroblasts in the knee joint.

Nuclear-factor-kappa B (NF-κB) is a transcriptional factor and closely related to the expression of cytokines and chemokines in initial inflammation. There have been some reports that described that PDT could directly activate NF-κB [46]–[48]. In our examination, however, NF-κB inhibitor (SN50) did not significantly decrease the effect of Pre-PDT (see Figure S3, indicating that signaling cascades other than NF-κB activation were probably responsible for the Pre-PDT effect. Further studies are needed to define in more detail exactly which signaling pathways are involved in the protective response against MRSA produced after PDT. In particular the role of the transcription factor AP-1 should be investigated and the exact role of IL6 needs to be explored.

The important (and sometimes crucial) role of neutrophils in the therapeutic response of tumors to various PDT regimens has been reported by several laboratories. De Vree et al [49] showed that depletion of neutrophils using a neutralizing antibody abrogated the tumoricidal effect of PDT, while increasing the number of circulating neutrophils with injection of granulocyte colony-stimulating factor potentiated the anti-tumor effect. Cecic et al [50] found a rapidly developing systemic (as well as local) neutrophilia in tumor-bearing mice after PDT with two different PS that could be abrogated by inhibitors of complement activation. These authors went on to show [51] that many mediators such as IL-1beta, TNF-alpha, IL-6, IL-10, G-CSF and KC, thromboxane, prostaglandins, leukotrienes, histamine, and coagulation factors were involved in this phenomenon and all were increased after complement activation after PDT. Although the role of PDT-activated/stimulated neutrophils in the therapeutic effects of PDT against cancer is established, the role of neutrophils in the therapeutic effects of antimicrobial PDT had not been previously reported before our studies in murine bacterial arthritis [33]–[35].

To apply Th-PDT or Pre-PDT using methylene blue as a therapeutic or preventive modality for clinical orthopedic microbial infections, several further challenges remain: the mechanism by which PDT using methylene blue potentiates neutrophil accumulation into the infected site needs to be elucidated; the development of a device that enables uniform and efficient light irradiation in an infected joint is required; more study of PS and light dosimetry would also be required; validation would be requited using a large animal model of orthopedic infection.

PDT may have some advantages compared with conventional antibiotic therapy: PDT could have a potential to be effective for infections caused by various bacteria regardless of antibiotic susceptibility; PDT could be applied as a preventive (prophylactic) strategy for a surgical-site infection after orthopedic surgery such as total knee arthroplasty, as well as a therapeutic modality for a traumatic or a post-surgical infection in orthopedics. PDT could be a new strategy for both the treatment and prevention of bone and joint bacterial infections more widely than the knee, as well as for intractable arthritis caused by multiple-drug-resistant bacteria.

In conclusion, therapeutic PDT using methylene blue exerted a promising therapeutic effect in a murine chronic MRSA arthritis model *via* neutrophil accumulation and migration. Preventive PDT used as a pre-conditioning regimen before bacterial inoculation suppressed the bacterial growth and inhibited the establishment of infection. This is the first demonstration of a protective innate immune response against a microbial pathogen being induced by PDT. This study did not evaluate the adaptive immune response (if any) induced after PDT for MRSA infection. Further studies should evaluate possible changes in Th-1, Th-2 and Th-17 T-cells and antibodies from B-cells.

## Materials and Methods

### Ethics Statement

This study was approved by the Subcommittee on Research Animal Care (IACUC) of Massachusetts General Hospital, USA (protocol 2005N000111) and the Institutional Review Board for the Care of Animal Subjects at the National Defense Medical College, Japan (protocols 08007 and 11008).

### Mouse Model of Intractable MRSA Arthritis Using Bioluminescent MRSA and Resin Microparticle

Stably bioluminescent MRSA Xen31 (Caliper Life Sciences, Alameda, CA: *lux*-MRSA) [18], [37] and resin microparticle (MP) made of polystyrene (Copolymer Microsphere Suspensions 7000 ϕ3.2 µm, Thermo Scientific Particle Technology, Fremont, CA) were used. The bacteria (*lux*-MRSA) were grown overnight in brain heart infusion (BHI) medium at 37°C with shaking at 100 rpm. Cell growth was assessed with an Evolution 300 UV-Vis Spectrophotometer (Thermo Scientific, Waltham, MA). When cultures reached an optical density (OD_600_) of 0.8, which corresponds to a bacterial cell density of 10^8^ CFU/mL, they were washed and resuspended in phosphate-buffered saline (PBS) (Dulbecco) at 1×10^10^ CFU/mL. Eight to nine-week-old male C57BL/6 mice (Charles River Laboratories, Wilmington, MA) were anesthetized by intraperitoneal (i.p.) injections of a cocktail composed of 100-mg/kg ketamine and 10-mg/kg xylazine and the left knees were shaved. According to the method described [33], [34], 10 µL of PBS suspension containing *lux*-MRSA (1×10^8^ CFU) and MP (2.5% volume) was intra-articularly injected into the left knee joint through the midline of the patellar ligament using a syringe with a 29 G needle (MP+ group). As a control, 10 µL of PBS suspension containing *lux*-MRSA (1×10^8^ CFU) but not MP was intra-articularly injected into the left knee joint (MP- group), and the severity of arthritis was compared with that of MP+ group.

### Evaluation of MRSA Arthritis by a Measurement of Bioluminescent Intensity

An image intensifier-equipped CCD photon-counting camera (Model C2400-30H; Hamamatsu Photonics, Bridgewater, NJ) mounted in a light-shielded specimen chamber, a computer system with Microsoft Windows 98 through an image processor (Argus-50, Hamamatsu Photonics) and an Argus-50 control program (Hamamatsu Photonics) was used to acquire images and to process the image data collected. As described [18], [19], [52], entire photon count of the left knee was quantified as relative luminescence units (RLUs) and was displayed in a false color scale ranging from pink (most intense) to blue (least intense). The bit range was fixed at 1–3 and the collecting time was fixed at 3 minutes.

### Photodynamic Therapy Using Methylene Blue

#### Therapeutic PDT (Th-PDT)

The day in which *lux*-MRSA and resin microparticle (MP) were inoculated into the knee joint was defined day 0 (0d). Twenty-four hours after the inoculation (at 1d), 10 µL of PBS solution of methylene blue (100 µM) was injected into the joint followed by immediate irradiation using a xenon light source with a bandpass filter (wavelength of 660±15 nm, LumaCare, Newport, CA). The fluence rate was fixed at 100 mW/cm^2^ and six different fluences ranging from 5 to 160 J/cm^2^ were used (5, 20, 50, 80, 120 and 160 J/cm^2^ in each group of mice, *n* = 5). Three other groups of mice were prepared as controls: (1) an IR- group, which received MB solution without subsequent photoirradiation, (2) a PS- group, which received photoirradiation without MB injection, (3) a PS-IR- group, which receive neither MB nor irradiation.

Bioluminescent intensity was measured 1 day after the inoculation of *lux*-MRSA with MP (at 1d), immediately after PDT (after PDT) and 2, 3, 5 and 7 days after the inoculation of *lux*-MRSA and MP (at 2d, 3d, 5d and 7d). The bioluminescent intensity value of each mouse at 1d was defined ‘1’ and the values of the other time points were expressed as relative values.

#### Preventive PDT (Pre-PDT)

The day in which *lux*-MRSA and resin microparticle (MP) were inoculated into the knee joint was defined day 0 (0d). Twenty-four hours (at −1d) or two hours (at −2h) before the inoculation, 10 µL of PBS solution of methylene blue (concentration of 100 µM) was injected into the joint followed by immediate irradiation using the light source in the fluence of 50 J/cm^2^ (a Pre-PDT (−1d) group and a Pre-PDT (−2h) group). Bioluminescent intensity was measured 2 hours (at 2h), 1, 2, 3, 5 and 7 days (at 1d, 2d, 3d, 5d and 7d) after the inoculation of *lux*-MRSA with MP. The bioluminescent intensity value of each mouse at 2h was defined ‘1’ and the values of the other time points were expressed as relative values.

### Follow-up of Leg Function and Local Erosion

The motility of each leg of the mice was evaluated for function and marked on the following scale 0–4 (leg function score) [19]: 4 =  perfectly normal leg in appearance and motion; 3 =  slight limp, slight impairment in movement; 2 =  significant impairment in movement, mouse cannot walk normally; 1 =  leg is paralyzed and dragged behind mouse; 0 =  legs suffers from frank necrosis or is absent. Additionally, their legs were observed for local erosion and marked on the following scale from 0–4 (erosion score): 4 =  no erosion with normal skin; 3 =  slight erythema or discolored skin; 2 =  skin necrosis with scabbing; 1 =  diffuse skin defect with exposure of subcutaneous tissue; 0 =  exposure of bone or absence of the leg.

### Histopathological Evaluation

#### Therapeutic PDT (Th-PDT)

Mice in the PS-IR- group, the 5, 50 and 160 J/cm^2^ group were sacrificed at 1d, 2d or 5d (*n* = 3). Knee joints were extracted and fixed with 10% formaldehyde solution for 48 hours and then decalcified with 10% ethylenediaminetetraacetic acid (EDTA)-2Na solution (pH 7) for 14 days. The tissue samples were then processed for hematoxylin-eosin (HE) staining and immunostaining for neutrophil-specific staining using a rat anti-mouse LY-6G/GR-1 antibody (SouthernBiotech, Birmingham, AL), a VECTASTAIN Elite ABC Kit (Vector Laboratories, Burlingame, CA) and a VECTOR NovaRED Substrate Kit (Vector Laboratories).

#### Preventive PDT (pre-PDT)

Mice in the Pre-PDT (−1d) group were sacrificed immediately after Pre-PDT (at −1d), just before MRSA inoculation with MP (at 0d), 2 hours (at 2h), 1 day (at 1d) and 5 days (at 5d) after inoculation (*n* = 3). Knee joints were processed as described above. Mice in the PS-IR- group were sacrificed at 0d ( =  normal knee joint), 2h, 1d and 5d and the knee joint was processed as described above.

### PDT Effect in Neutrophil-depletion Models

#### Therapeutic PDT (Th-PDT)

Mice in the 50 J/cm^2^ group were administrated rat anti-mouse LY-6G/GR-1 antibody (4 mg/kg at 1d, 2 mg/kg at 2d and 2 mg/kg at 3d) by an intravenous injection [53] and time course-bioluminescent intensity of the knee joint was evaluated (*n* = 5).

#### Preventive PDT (Pre-PDT)

Mice in the Pre-PDT −1d group were received rat anti-mouse LY-6G/GR-1 antibody (4 mg/kg at −1d, 2 mg/kg at 0d and 2 mg/kg at 1d) by an intravenous injection and time course-bioluminescent intensity of the knee joint was evaluated (*n* = 5).

### Time Courses of Intra-articular Leukocyte Counts after Preventive PDT (Pre-PDT)

Two microliters of synovial fluid was collected from the knee joint of a mouse in the Pre-PDT (−1d) group, and the numbers of leukocytes in the synovial fluid at −1d, 0d, 2h, 1d or 5d were estimated by the methods described previously [33], [34]. The numbers of intraarticular leukocytes in the PS-IR- group at 0d, 2h, 1d or 5d were also estimated by the same methods (*n* = 5 in each group).

### Neutralization of Chemotactic Factors in the Preventive PDT (Pre-PDT)

Involvement of each chemotactic factor was evaluated using neutralizing antibody [53]. Mice in the Pre-PDT (−1d) group were administrated each antibody described below by an intravenous injection (4 mg/kg at −1d, 2 mg/kg at 0d and 2 mg/kg at 1d), and the bioluminescent intensity of the knee joint was sequentially evaluated: a mouse anti-mouse interleukin 1-beta (IL-1β) antibody (Thermo Scientific Pierce, Rockford, IL), a goat anti-mouse tumor necrosis factor alpha (TNF-α) antibody (Sigma-Aldrich), a rabbit anti-mouse interleukin 6 (IL-6) antibody (Thermo Scientific Pierce), a goat anti-mouse macrophage inflammatory protein 2 (MIP-2) antibody (Sigma-Aldrich), a rabbit anti-keratinocyte-derived chemokine (KC) antibody (Thermo Scientific Pierce), a hamster anti-mouse intercellular adhesion molecule 1 (ICAM-1) antibody (BD Biosciences, San Diego, CA) and a rat anti-mouse E-selectin antibody (BD Biosciences) (*n* = 5 in each group).

In addition, mice in the Pre-PDT (−1d) group were given SN50 [38] (Enzo LifeSciences, Plymouth Meeting, PA), which is an inhibitor of nuclear factor-kappa B (NF-κB), one of the transcription factor for initial inflammatory reaction, by an intravenous injection (4 mg/kg at −1d, 2 mg/kg at 0d and 2 mg/kg at 1d), and the bioluminescent intensity of the knee joint was sequentially evaluated (*n* = 5). Integral values of the time courses of bioluminescent intensity at from 2h to 7d were calculated and expressed as the area under the curve (AUC). AUC in each group were statistically compared to that in the PS-IR- group as described previously [18].

### Statistical Analysis

All the data are expressed as means ± SE. Mean values in each group or AUC were used for statistical analysis. One-way repeated-measures analysis of variance (ANOVA) test followed by Dunnett's post *hoc* test was used for data analysis of time courses. Two-way repeated-measures ANOVA test followed by paired *t*-test was used for data analysis of intraarticular leukocyte counts. One-way ANOVA test followed by Dunnett's post *hoc* test was used for data analysis of AUC. SPSS ver.16 was used for each data analysis. *P*-values <0.05 were considered statistically significant.

## Supporting Information

Figure S1 **Leg function and erosion scores after Th-PDT.** a: Time courses of the leg function score after therapeutic PDT (Th-PDT) in each irradiation energy group. b: Comparison of the area under the curve (AUC) of the data indicated in b. c: Time courses of the erosion score. d: Comparison of the area under the curve (AUC) of the data indicated in c. n = 5 each. *P<0.05, **P<0.01.
(TIFF)

Figure S2 **Leg function and erosion scores after Pre-PDT.** a: Time courses of the leg function score after preventive PDT (Pre-PDT) in each group. b: Comparison of the area under the curve (AUC) of the data indicated in b. c: Time courses of the erosion score. d: Comparison of the area under the curve (AUC) of the data indicated in c. n = 5 each. **P<0.01.
(TIFF)

Figure S3 **Effect of neutralizing antibodies on bioluminescence in Pre-PDT.** Serial time courses of the bioluminescent intensity in each Pre-PDT group using neutralizing antibodies for chemotactic factors.
(TIFF)
