# Supplementary figures and images for: Photodynamic Therapy Can Induce a Protective Innate Immune Response against Murine Bacterial Arthritis via Neutrophil Accumulation
Source: PLoS One. 2012 Jun 26;7(6):e39823. doi: 10.1371/journal.pone.0039823 (PMC3383702; doi:10.1371/journal.pone.0039823)

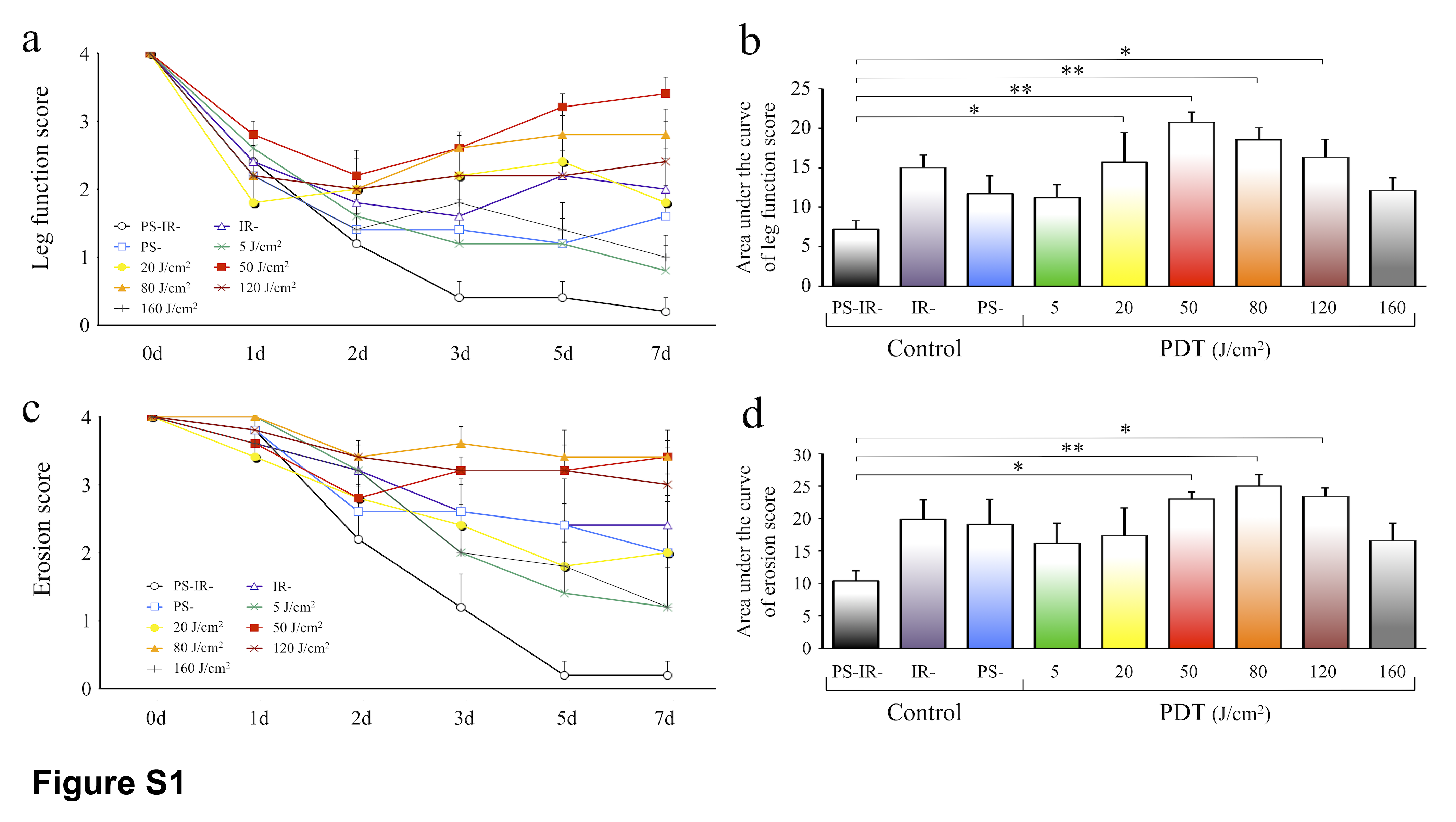

Supplement: Figure S1 — Leg function and erosion scores after Th-PDT. a: Time courses of the leg function score after therapeutic PDT (Th-PDT) in each irradiation energy group. b: Comparison of the area under the curve (AUC) of the data indicated in b. c: Time courses of the erosion score. d: Comparison of the area under the curve (AUC) of the data indicated in c. n = 5 each. *P<0.05, **P<0.01. (TIFF) [file pone.0039823.s001.tiff]

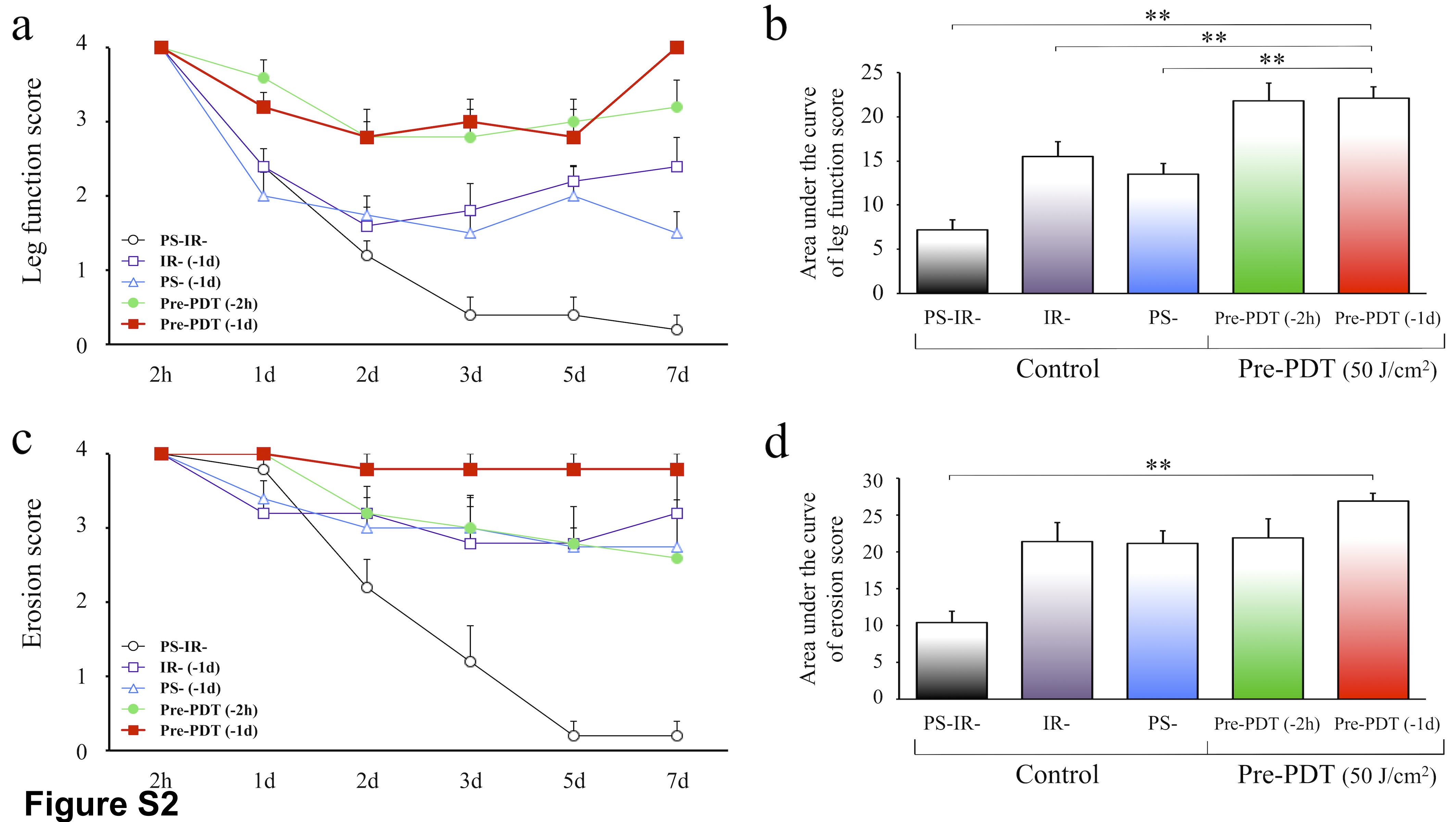

Supplement: Figure S2 — Leg function and erosion scores after Pre-PDT. a: Time courses of the leg function score after preventive PDT (Pre-PDT) in each group. b: Comparison of the area under the curve (AUC) of the data indicated in b. c: Time courses of the erosion score. d: Comparison of the area under the curve (AUC) of the data indicated in c. n = 5 each. **P<0.01. (TIFF) [file pone.0039823.s002.tiff]

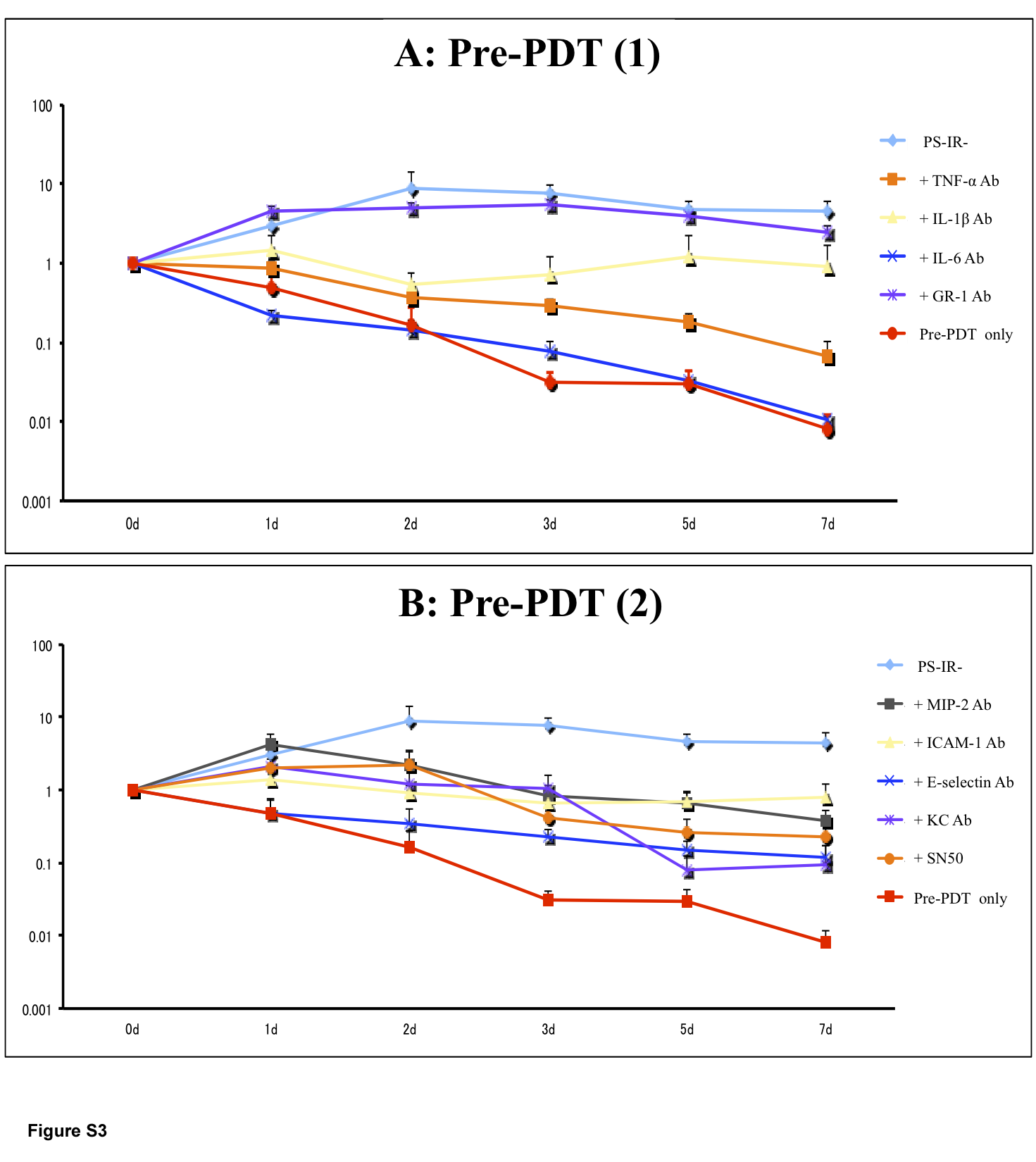

Supplement: Figure S3 — Effect of neutralizing antibodies on bioluminescence in Pre-PDT. Serial time courses of the bioluminescent intensity in each Pre-PDT group using neutralizing antibodies for chemotactic factors. (TIFF) [file pone.0039823.s003.tiff]
